# Identification, characterization, and utilization of single copy genes in 29 angiosperm genomes

**DOI:** 10.1186/1471-2164-15-504

**Published:** 2014-06-21

**Authors:** Fengming Han, Yong Peng, Lijia Xu, Peigen Xiao

**Affiliations:** Institute of Medicinal Plant Development, Chinese Academy of Medical Sciences, Beijing, 100193 PR China

**Keywords:** Single copy gene, Duplication, Gene Ontology, Codon usage, GC3, Gene expression, *Ka*/*Ks*, Alternative splicing, Phylogeny

## Abstract

**Background:**

Single copy genes are common across angiosperm genomes. With the sufficiently high quality sequenced genomes, the identification of large-scale single copy genes among multiple species is possible. Although some characteristics have been reported, our study provides novel insights into single copy genes.

**Results:**

We identified single copy genes across 29 angiosperm genomes. A significant negative correlation was found between the number of duplicate blocks and the number of single copy genes. We found that a considerable number of single copy genes are located in organelles, showing a preference for binding and catalytic activity. The analysis of effective number of codons (Nc) illustrates that single copy genes have a stronger codon bias than non-single copy genes in eudicots. The relative high expression level of single copy genes was partially confirmed by the RNA-seq data, rather than the Codon Adaptation Index (CAI). Unlike in most other species, a strongly negatively correlation occurs between Nc and GC3 among single copy genes in grass genomes. When compared to all non-single copy genes, single copy genes indicate more conservation (as indicated by *Ka* and *Ks* values). But our alternative splicing (AS) results reveal that selective constraints are weaker in single copy genes than in low copy family genes (1–10 in-paralogs) and stronger than high copy family genes (>10 in-paralogs). Using concatenated shared single copy genes, we obtained a well-resolved phylogenetic tree. With the addition of intron sequences, the branch support is improved, but striking incongruences are also evident. Therefore, it is noteworthy that inclusion of intron sequences seems more appropriate for the phylogenetic reconstruction at lower taxonomic levels.

**Conclusions:**

Our analysis provides insight into the evolutionary characteristics of single copy genes across 29 angiosperm genomes. The results suggest that there are key differences in evolutionary constraints between single copy genes and non-single copy genes. And to some extent, these evolutionary constraints show some species-specific differences, especially between eudicots and monocots. Our preliminary evidence also suggests that the concatenated shared single copy genes are well suited for use in resolving phylogenetic relationships.

**Electronic supplementary material:**

The online version of this article (doi:10.1186/1471-2164-15-504) contains supplementary material, which is available to authorized users.

## Background

Duplication events (e.g. whole genome duplication, segmental duplication, local and tandem duplication) are prevalent through the evolutionary history of flowering plants [1]. These duplications are potential sources of genes with new functions, thus contributing to taxonomic diversity. However, some duplicates might be loss as the protein or transcriptional network balance is maintained [2–5]. In certain situations, some genes are actually returned to a single copy status. In general, single copy genes may occur in two ways following duplicastions: (1) a neutral process in which dosage-insensitive duplicate genes are not under selection to lose randomly in the genome, and (2) the other is involved in selection that dosage-sensitive genes are more likely to repeatedly restore to a single copy, occurring as duplication-resistant [6–8]. Using comparative genomics, several studies have focused on characterization of single copy genes in plants. In earlier studies, single copy genes were analyzed only in a few genomes (e.g. *Arabidopsis*, *Populus*, *Vitis* and *Oryza*) [9, 10], thus there remains a lack of understanding about many of the evolutionary and functional characteristics of such genes. De Smet et al. [11] were the first to investigate the existence of single copy genes in 20 flowering plants, and from that study proposed that a subset of these single copy genes were under strong selective pressure to remain as a singleton. In addition, these single copy genes were highly conserved and were found to participate in essential housekeeping functions. However, the authors did not further examine the underlying species-specific differences related to single copy genes. In fact, following the lineage divergence, the mechanisms by which single copy genes having been experienced birth and death within different lingages might be diverse, thereby making characterizing the single copy genes complicated.

Traditionally, single copy genes were primarily used as markers in plant systematic studies, (e.g. *waxy*, *leafy*, *alcohol*, *nepGS*, *GIGANTEA*, *GPA1*) [12–14]. Compared with use of rDNA sequences, such are not subject to concerted evolution and thus facilitate homologous comparison [12]. Perhaps more importantly, there are a large number of single copy genes in plant genomes, thus providing wide range of markers. The single copy genes listed above were first identified in a single genome, thus when used to phylogenetic reconstruction at a higher taxonomic level, it was not clear if there is a paralog in other genomes. It is therefore necessary to identify a set of single copy genes shared by multiple genomes to better resolve these phylogenetic trees. Similar studies have been completed in *Arabidopsis*, *Populus*, *Vitis* and *Oryza* [9, 10]. However, the number of genomes investigated was too low, and attention was focused only on protein-coding sequences, without inclusion of intron sequences. This is of importance as intron regions are often used to explain some phylogenetic hypotheses. For example, the 7th to 10th introns of the nepGS gene (Chloroplast-Expressed Glutamine Synthetase) from *Oxalis* (Oxalidaceae) are used as a phylogenetic marker at lower taxonomic levels [15]. It is therefore important to further evaluate the effects of intron on phylogeny reconstruction when using single copy genes.

In this present study, single copy genes were identified and characterized from the previously sequenced genomes of 29 angiosperms. Codon choice may affect various molecular mechanisms, including protein folding, exon splicing, translational accuracy and efficiency [16–19]. Thus to evaluate codon bias, codon usage indices were measured among the single copy genes. In addition, the selective constraints imposed on single copy genes were also examined. We also assessed the reliability of phylogeny developed from identified single copy genes.

## Results

### Identification of single copy genes

The percentage of single copy genes identified in 29 angiosperm genomes ranged widely, from approximately 8%-35% (details in Table 1). The lower percentage was for *Brassica rapa* and *Glycine max*, while the higher was for *Ricinus communis* and *Carica papaya*. When collinear genes were examined within each lineage, there was a significant and negative correlation between the number of duplicate blocks and the number of single copy genes (Spearman test, *r* = -0.694, *p* = 2.956e-005). In each genome there were a number of species-specific singletons. The ratio (singleton/total single copy) was between approximately 10%-68%. There was no significant correlation between the number of duplicate blocks and the number of singletons. However, we did note that an increase in genome number was associated with a decrease in the number of shared single copy genes among different lineages. The identified single copy genes (including the number of BLAST hits) across 29 angiosperm genomes are listed in Additional file 1.

**Table 1:** **Identification of single copy genes and the predicted replicate blocks within each lineage**

Species	Total number of genes	Number of single copy genes	Singletons	Number of replicate blocks
*Aquilegia coerulea*	24823	4962	1886	65
*Arabidopsis lyrata*	32670	4863	1710	89
*Arabidopsis thaliana*	27382	4285	919	211
*Brachypodium distachyon*	26552	3987	733	151
*Brassica rapa*	41019	3098	1366	745
*Capsella rubella*	26521	3613	393	179
*Carica papaya*	27584	8292	3377	60
*Citrus clementina*	24533	4550	876	131
*Citrus sinensis*	25379	4883	946	111
*Cucumis sativus*	21458	4778	1309	149
*Eucalyptus grandis*	36376	6897	3930	316
*Fragaria vesca*	32831	6132	3547	122
*Glycine max*	45272	3687	2348	777
*Gossypium raimondii*	37505	3823	1456	801
*Manihot esculenta*	30666	3788	672	476
*Mimulus guttatus*	26718	4081	724	266
*Oryza sativa*	55565	9995	6829	160
*Phaseolus vulgaris*	27197	3760	554	342
*Populus trichocarpa*	40668	4435	2502	544
*Prunus persica*	27864	3922	689	122
*Ricinus communis*	31221	11095	7206	115
*Setaria italica*	35471	7608	4130	154
*Solanum lycopersicum*	34727	6274	2687	317
*Solanum tuberosum*	35119	5665	2388	238
*Sorghum bicolor*	33032	6761	3399	152
*Thellungiella halophila*	26351	3676	511	177
*Theobroma cacao*	29408	7226	3434	139
*Vitis vinifera*	26346	5684	2501	143
*Zea mays*	39656	6184	3307	383

The singleton is referred as a single copy gene which no orthologs are found in other lineages.

### Gene Ontology (GO) enrichment analysis of single copy genes

**Figure 1:**
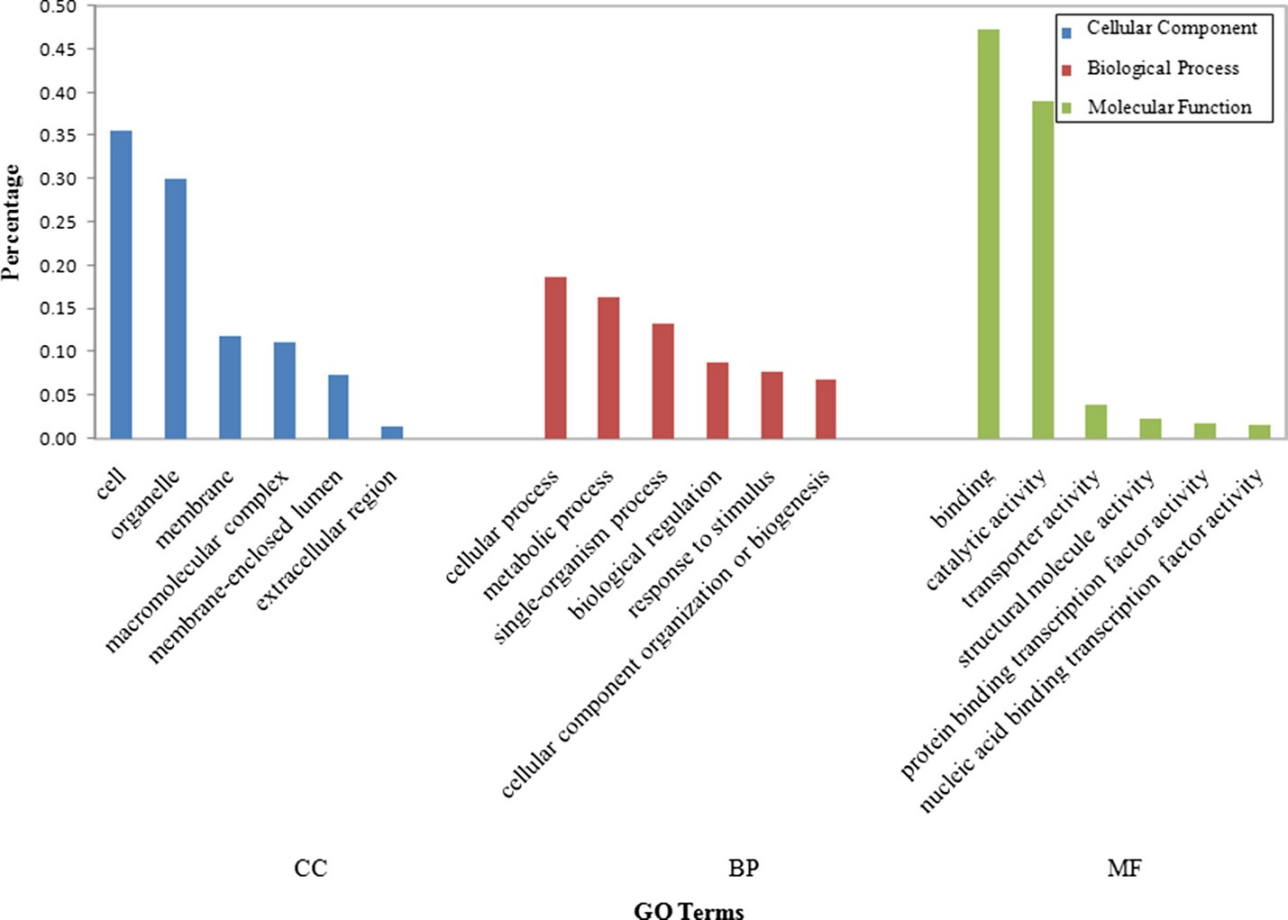
**GO term enrichments of united all single copy genes across 29 angiosperm genomes.** The vertical axis shows the percentage of annotated sequences within each GO categories. Only the top six GO terms are showed. CC: Cellular Component; BP: Biological Process; MF: Molecular Function.

We conducted GO enrichments of species-specific single copy genes and the united all single copy genes in all 29 species. The top two enriched GO terms in the three GO categories (i.e. ‘Cellular Component’, CC; ‘Biological Processes’, BP; ‘Molecular Function’, MF) were consistent among individual species and united across all the 29 species (Figure 1). In the CC terms, the top two enriched GO terms were related to cell and organelle, the BP terms were related to cellular and metabolic processes, and the MF terms were related to binding and catalytic activity. Details of the high level of the function enrichment of single copy genes were in the Additional file 2.

### Effective number of codons (Nc)

The Nc values [20] were computed to characterize codon usage bias in single copy genes. This measure is independent of gene length and amino acid composition and ranges from 20 (an extreme bias) to 61 (equally use synonymous codons). We found that single copy genes in 22 eudicot species showed stronger codon bias than non-single copy genes (with the exception of *Prunus persica* and *A. lyrata* (Additional file 3). However, the opposite was true for *Sorghum bicolor*, *Zea mays*, *Setaria italica*, *O. sativa* and *Brachypodium distachyon*, all grass species.

### Expression level, Codon Adaptation Index (CAI) and GC content at the third positions in codons (GC3)

RNA-seq data obtained from four species were used to examine differences in expression levels. The results were consistent with that obtained in earlier studies in that high expression levels were found in the single copy genes (Additional file 4). However, the CAI values (a gene transcriptional index) [21, 22] was inconsistent with the expression levels in *O. sativa* and *Z. mays* where higher CAI values occurred among non-single copy genes (Additional file 5). Several studies indicated that there is either a weak or strong negative correlation between gene expression level and GC3 content [23–27]. Our results partially revealed that the GC3 values were significantly lower for single copy genes than for non-single copy genes in the 22 plant species examined (Additional file 3).

### Alternative splicing (AS)

**Figure 2:**
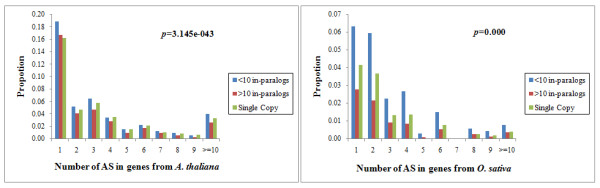
**Distribution of alternative splicing (AS) events between single copy genes and other family genes.** The vertical axis represents the proportion of genes for which AS is found. The horizontal axis represents the different numbers of AS. The significance of AS differences between single copy genes and family genes is calculated by the use of the analysis of variance (ANOVA) with subsequent post-hoc tests (Tukey’s HSD).

Alternative splicing is a major factor in increasing species diversity and regulatory complexity [28], and has significant effects on the evolution of coding exons [29, 30]. To assess the AS difference between single copy genes and non-single copy genes, AS data sets from different growth and developmental stages, or multiple organs of *A. thaliana* and *O. sativa* were analyzed. Single copy genes had on average, increased levels of AS relative to gene families containing at least 10 in-paralogs. In contrast, those gene families with 1–10 in-paralogs had a higher proportion of AS than did single copy genes (Figure 2).

### Phylogeny reconstruction using single copy genes

**Figure 3:**
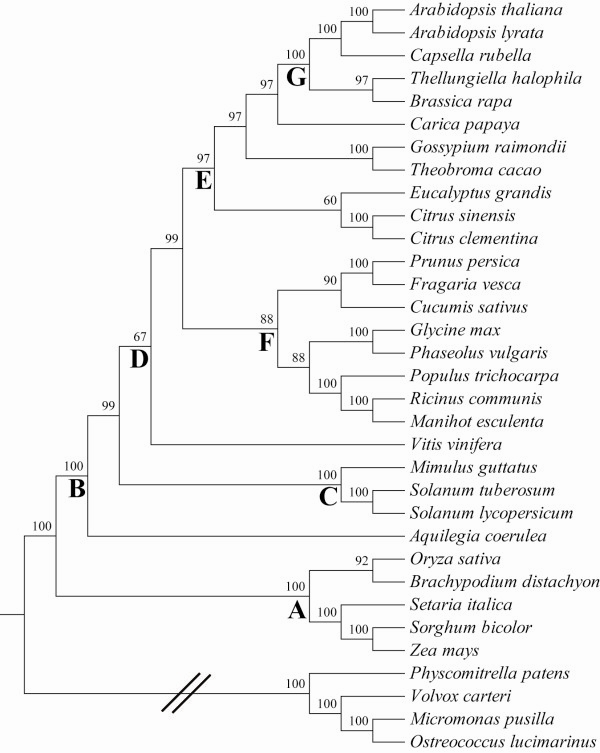
**Maximum likelihood (ML) phylogenetic tree bases on the 12 shared single copy genes.** Numbers above nodes are bootstrap proportions from 1000 pseudoreplicates. *O. lucimarinus*, *M. pusilla*, *V. carteri* and *P. patens* were used as outgroups. The letters embedded below nodes: A = Grass; B = Eudicots; C = Asterids; D = Rosids; E = Malvidae; F = Fabidae; G = Brassicaceae. The double slashes indicate outgroups.

Twelve shared single copy genes of 29 angiosperm genomes were used to evaluate the power of phylogenetic reconstruction. Maximum likelihood phylogenetic trees were reconstructed using individual and concatenated twelve shared single copy genes. When an individual single copy gene was used, relatively high incongruence was observed among the individual single copy genes, except that the grass species possessed consistent topology with high bootstrap values (data not shown). The concatenated twelve shared single copy genes recovered an identical topology with proposed by APG III [31] (Figure 3).

To assess the role of introns in phylogenetic reconstruction, a second set of phylogenetic trees were recovered using single copy genes with the inclusion of introns. Again, high incongruence was observed among the individual single copy genes. Similarly, grass species still recovered well with high bootstrap values (data not shown). The concatenated twelve shared single copy genes recovered a fully resolved topology for all 29 plant species, with 100% bootstrap values for every node except for one (Additional file 6). However, there were some artificial branches generated by the presence of introns. For example, the *V. vinifera* was sister to *P. vulgaris* and *G. max* other than rooting the large rosids group [31] and on the other side, *Gossypium raimondii* and *Theobroma cacao* in the Malvidae group clustered with the Fabidae group with a 100% bootstrap value.

## Discussion

### Varied number of single copy genes within angiosperm genomes

De Smet et al. [11] have previously reported single copy genes in 20 angiosperm genomes, but they focused on the conserved single copy genes across multiple lineages other than characterize the species-specific single copy genes. Additionally, those genes with duplicates in up to three species can be regarded as single copy genes, thus the number of shared single copy genes may be higher than the species-specific single copy genes. For example, there were 14993 shared single copy genes between *A. thaliana* and *A. lyrata* according to previous reports but we have only identified 4285 and 4863 single copy genes in those two species, respectively. In our study, large variance in the number of single copy genes within angiosperm genomes was found. This copy number divergence was partly attributed to duplication events within each genome. In particular, whole genome duplication can lead to an instantaneous change in gene content. If single copy genes were present as a result of selective pressure, one copy would inevitably be lost in the subsequent evolutionary step. It is not clear what mechanism(s) dictate which single copy genes will remain, and which will be lost. The negative correlation between the number of duplicate blocks and the number of single copy genes indicated that duplicate events greatly impact the number of single copy genes, so some homologous single copy genes may not be equally restored to single copy status, due to different duplication frequencies across different lineages. In addition, the early duplicate cues can be disrupted by the unequal crossing-over, gene conversion, retroposition, etc., thus complicating the relationship between the duplicate events and the single copy genes [1, 32–34]. Moreover, except for duplication-resistant genes, other duplicate genes may randomly revert to single copy status as dosage-insensitive genes are more likely to exhibit copy number variation [35]. Therefore, it was not entirely surprising that the number of shared single copy genes decreased as more genomes were compared.

### Characteristics of single copy genes

The cell and organelle location indicated that a large proportion of single copy genes coded for intracellular and organelle components. This suggested that single copy genes may be involved in a cascade of processes in which single copy genes are first coded in nuclei, and then transported to organelles where single copy genes are very common. In order to maintain proper stoichiometric balance with interacting partner(s) that are encoded in the organelle genome, single copy genes coded in nuclear genome may tend to remain in a single copy status [9]. It is noteworthy that one of the most enriched MF terms of single copy genes were related to binding functions. Thus, many single copy genes may participate in the formation of macromolecular complexes to maintain essential metabolic processes. Because those complexes are sensitive to the stoichiometric balance [7], single copy genes would need to be duplication-resistant.

Codon usage bias can be used to analyze selective pressure operating on genes. Based on the Nc values, there was a strong codon bias for single copy genes than for non-single copy genes in eudicots. In addition, there was a weaker correlation between Nc and GC3 values for single copy genes than for non-single copy genes (Additional file 7). This may provide indirect evidence that translational selection more strongly dictates codon usage bias other than compositional bias for single copy genes in eudicots. There was more strongly negative correlation between the Nc values and the GC3 values in grass species. Thus, relative to that in eudicot species, more compositional bias may occur among the single copy genes in monocot species. In addition, in light of the differential CAI indices, we estimated that some single copy genes follow distinct evolutionary routes after eudicot-monocot divergence. Strong evidence for this hypothesis is that eudicot species share a common genome triplication, different from monocot species [36–38]. Previous studies also provided direct evidences that eudicot and monocot species share different features of codon usage [39–41]. We concluded that a clear distinction among codon usage of single copy genes between eudicot and monocot species was evident and beneficial to experimental verification in the future.

The GC3 values tended to be lower in single copy genes than in non-single copy genes indicating higher gene expression levels, consistent with that observed in earlier studies. This was also confirmed in part by the RNA-seq analysis from the *O. sativa*, *Z. mays*, *P. vulgaris* and *G. max* in our study and previous study of De Smet et al. [11] which based on expression levels of single copy genes in *A. thaliana*. Therefore, single copy genes might be under stronger selective constraints [11]. But the relationship between gene expression level and GC3 content needs to be further validated. Similar to previous studies, the more conservative evolution of single copy genes was supported by the values of *Ka* and *Ks* in two species pairs, *A. thaliana* vs *G. max* and *Solanum tuberosum* vs *S. lycopersicum,* which represented different taxonomic levels in our study (Additional file 8). Our results revealed that AS events were significantly correlated with the gene number of gene family and that single copy genes had on average, a lower number of AS events relative to low copy family genes. According to earlier reports [29, 42], this suggested that AS may impose a stronger selective constraint on low copy family genes than on single copy genes. Previous studies found that AS and gene duplication did not evolve independently [43, 44]. The gene duplication can result in the loss of AS because the functions of different AS isoforms may be kept by different duplicate genes. The large family genes tend to have less AS due to multiple rounds of gene duplication, but the ancient duplicate gene may undergo neofunctionalization by acquiring new functional AS isoforms. We speculated that a large set of small gene family may experience the loss of in-paralogs and the functions of former in-paralogs may be complemented by the new AS isoforms of remaining gene copies, thus showing higher level of AS. Furthermore, Ner-Gaon et al. [45] found that the increase of genome size can lead to the additional AS increment in eudicots other than in monocots because the expansion of genomes in monocots was mainly due to the nested retrotransposons within noncoding regions other than within genes. Taken together, a more detailed investigation of species specificity, evolutionary mechanism specificity, and function specificity corresponding to single copy genes should be undertaken in the future.

### Phylogenetic reconstruction using single copy genes

The use of molecular characters provides a wealth of new information that sheds light on many components of the plant tree of life. The advantage of the single copy genes is their bi-parental inheritance (compared to maternally inherited plastid markers), and the absence of paralogs [46, 47]. These features are useful for use in studies aimed at unraveling patterns of reticulate evolution, hybrid formation, and parentage of polyploids [48]. In our study, the use of individual single copy genes was not able to resolve a clear phylogenetic tree in most instances. This may be due in part to: (1) a scarcity of informative characters, and (2) the incongruence between different single copy genes. This might be remedied by analysis of concatenated multiple shared single copy genes. With the addition of the intron sequences, the phylogenetic tree was fully resolved. But if it were true, several evolutionary pathways among plant kingdom would require major modification. So such a phylogenetic tree was unreasonable. One explanation for this contradiction was that the systematic error leading to tree reconstruction artifacts was produced due to the presence of a huge number of non-phylogenetic signals in intron sequences [49–51]. These systematic errors would not only average out by addition of number of sites but can dominate the true phylogenetic signal [51]. Therefore, caution should be followed when intron sequences are included in single copy genes, especially at higher taxonomic levels. Thus, we suggest that introns within single copy genes will be more specially suited to recover the relationships of intra-species.

## Conclusions

The number of single copy genes varied greatly within angiosperm genomes, and these genes may be involved in essential metabolic processes. In general, single copy genes in eudicots had a stronger codon usage bias than in monocots. The higher gene expression level and more conservative sequences suggested that single copy genes have a preferential role in biological pathways. But the single copy genes did not always exhibit a higher CAI value. So based on complex evolutionary mechanisms of single copy genes, there were also some species-specific characteristics among different lineages. As for the AS events, the selective constraints of single copy genes were stronger than for high copy family genes, but lower than low copy family genes, suggesting a moderate selective constraint. In view of our phylogenetic test, single copy genes are good sources for molecular markers to complement data from chloroplast sequences and rDNA, yet the inclusion of intron sequences may produce artifacts.

## Methods

### Twenty-nine genomic data sets of angiosperm genomes

Twenty-nine genomic data sets (including gene sequences, translated peptide sequences and coding sequences) were obtained from Phytozome (Phytozome v9.1, http://www.phytozome.net/), and PlantGDB (http://www.plantgdb.org/).

### Selecting single copy genes

To identify single copy genes in each species, each of the 29 protein data sets was compared against itself using BLAST program (ftp://ftp.ncbi.nlm.nih.gov/blast/executables/blast+/2.2.28/), with an e-value cutoff of 1e-10 and up to 200 alignments. Single copy genes were then extracted within each species by the tcl script (http://cgpdb.ucdavis.edu/BlastParser/Blast_Parser.html). There were three steps for identifying shared single copy genes: (1) all protein data sets were compared against each other using an all-blast-all BLAST with an e-value cutoff of 1e-5, coverage of at least 70% of the query protein and identity of at least 30%. Using MCL (The Markov Cluster Algorithm) method [52], (2) all protein sequences were clustered into different groups with a conservative and stringent inflation value of 5.0, and lastly since the outputs of MCL might be susceptible to the choice of the inflation parameter, determining the cluster tightness (granularity) [52, 53] (3) those roughly shared protein groups were simultaneously confirmed by multiple rounds of mutual blast procedures and using tcl script listed in above. According to the final clusters of orthologous genes, the number of BLAST hits of all species-specific single copy genes in 29 plant lineages was estimated. Identification of duplicate regions for each of the 29 angiosperm genomes was accomplished using MCScanX [54]. The parameters setting for MCScanX was as follows: the BLASTP was applied to find intra-species paralogous pairs with an e-value cutoff of 1e-10, and the duplicate blocks involved at least 5 collinear gene pairs and the gap gene pair number was not more than 20.

### Annotation enrichment

Single copy genes were annotated, by performing BLAST using the following reference databases: UniProtKB, RefSeq Protein and Ensembl Transcripts with an e-value cutoff of 1e-5. Then, GO terms associated to each single copy gene were analyzed using Blast2GO [55, 56]. Lastly, the frequency distributions of annotated single copy genes at multi-levels in three GO categories (CC, BP and MF) were calculated.

### Codon usage bias analysis

The values of Nc and GC3 were calculated with CodonW [57]. To quantify the CAI, a set of species-specific preferred codons (i.e. a set of highly expressed genes) must be prepared. The CAI indices of *O. sativa*, *Z. mays*, *P. vulgaris* and *G. max* were calculated using DAMBE where these species-specific preferred codons were built-in [58].

### Calculation of *Ka*and *Ks*

Pairs of homologous gene sequences were extracted from the best BLAST hit between two pairs of species, *A. thaliana* vs *G. max* and *S. tuberosum* vs *S. lycopersicum*. Each of these homologous pairs was aligned using MAFFT v7.122 [59]. The *Ka* and *Ks* were estimated for each homologous pair, and averaged over the entire alignment, using the KaKs Calculator v1.2 [60]. This program implements several candidate models of codon substitution in a maximum likelihood framework; we used the GY method to estimate *Ka* and *Ks* values.

### Expression levels and estimation of AS events

To estimate expression differences between single copy genes and non-single copy genes in *O. sativa*, *Z. mays*, *P. vulgaris* and *G. max*, data were retrieved from Rice Genome Annotation Project (http://rice.plantbiology.msu.edu), Crop Science Society of America (https://www.crops.org), Phytozome (http://www.phytozome.net) and Soybase (http://www.soybase.org/), respectively. The AS data exploration for different family genes referred to [61] and [62]. These two AS dataset included all the common types of AS events in *A. thaliana* (Col-0) and *O. sativa* (*O.sativa* L. ssp. *indica* cv. 9311), respectively. The analysis of variance (ANOVA) with subsequent post-hoc tests (Tukey’s HSD) was used to calculate the differences of AS between single copy genes and family genes. The tests were conducted on the values of proportions of genes with different numbers of AS isoform.

### Phylogeny reconstruction

Phylogenetic analyses and their corresponding bootstrap analyses were performed on the single copy genes, using the maximum likelihood (ML) method using RAxML version 7.6.3 [63] with 1000 rapid bootstrap analyses followed by a search of the best-scoring ML tree. These analyses were done using the CIPRES portal (http://www.phylo.org/portal2/home.action). As recommended by Stamatakis et al. [64], the general time reversible model was used with an alpha parameter for the shape of the gamma distribution to account for among-site rate heterogeneity for the datasets. In all phylogenetic trees, *Ostreococcus lucimarinus*, *Micromonas pusilla*, *Volvox carteri* and *Physcomitrella patens* were used as out-groups.

### Availability of supporting data

The phylogenetic trees supporting the results of this article are available at the Labarchives repository, DOI:10.6070/H49K486M, https://mynotebook.labarchives.com/share/hfm2014/MjIuMXwzODkwNy8xNy9UcmVlTm9kZS8xMzk4OTAzNXw1Ni4x.

## Electronic supplementary material

Additional file 1: **Identified single copy genes and their number of BLAST hits among 29 angiosperm genomes.** (XLSX 19 MB)

Additional file 2: **GO enrichments (3th level GO terms) of single copy genes in 29 angiosperm genomes.** CC: Cellular Component; BP: Biological Process; MF: Molecular Function. (XLSX 24 KB)

Additional file 3: **Mann–Whitney** ***U*** **test for effective number of codons (Nc) and GC3.** (DOCX 15 KB)

Additional file 4: **Average log expression levels for single copy genes in** ***O. sativa*** **,** ***Z. mays*** **,** ***P. vulgaris*** **and** ***G. max.*** The significance between differences is calculated by the use of Mann–Whitney *U* test. (TIFF 4 MB)

Additional file 5: **Codon Adaptation Index (CAI) for single-copy genes and no-single copy genes in** ***O. sativa*** **,** ***Z. mays*** **,** ***P. vulgaris*** **and** ***G. max.*** The significance between differences is calculated by the use of Mann–Whitney *U* test. (TIFF 4 MB)

Additional file 6: **The ML phylogenetic tree bases on the 12 shared single copy genes including introns.** Numbers above nodes are bootstrap proportions from 1000 pseudoreplicates. *O. lucimarinus*, *M. pusilla*, *V. carteri* and *P. patens* are used as outgroups. The letters embedded below nodes: A = Grass; B = Eudicots. The double slashes indicate outgroups. (ZIP 202 KB)

Additional file 7: **Spearman correlation test comparing effective number of codons (Nc) versus GC3.** (DOCX 15 KB)

Additional file 8: **Average values of** ***Ka*** **and** ***Ks*** **for single copy genes in two species pairs.** The significance between differences is calculated by the use of Mann–Whitney *U* test. (TIFF 4 MB)

## References

[1] Zhang J (2003). Evolution by gene duplication: an update. Trends Ecol Evol.

[2] Birchler JA, Newton KJ (1981). Modulation of protein levels in chromosomal dosage series of maize: the biochemical basis of aneuploid syndromes. Genetics.

[3] Song K, Lu P, Tang K, Osborn TC (1995). Rapid genome change in synthetic polyploids of Brassica and its implications for polyploid evolution. Proc Natl Acad Sci U S A.

[4] Shaked H, Kashkush K, Ozkan H, Feldman M, Levy AA (2001). Sequence elimination and cytosine methylation are rapid and reproducible responses of the genome to wide hybridization and allopolyploidy in wheat. Plant Cell.

[5] Papp B, Pal C, Hurst LD (2003). Dosage sensitivity and the evolution of gene families in yeast. Nature.

[6] Birchler JA, Riddle NC, Auger DL, Veitia RA (2005). Dosage balance in gene regulation: biological implications. Trends Genet.

[7] Edger PP, Pires JC (2009). Gene and genome duplications: the impact of dosage-sensitivity on the fate of nuclear genes. Chromosome Res.

[8] Makino T, McLysaght A (2010). Ohnologs in the human genome are dosage balanced and frequently associated with disease. Proc Natl Acad Sci U S A.

[9] Paterson AH, Chapman BA, Kissinger JC, Bowers JE, Feltus FA, Estill JC (2006). Many gene and domain families have convergent fates following independent whole-genome duplication events in *Arabidopsis*, *Oryza, Saccharomyces* and *Tetraodon*. Trends Genet.

[10] Duarte JM, Wall PK, Edger PP, Landherr LL, Ma H, Pires JC, Leebens-Mack J, de Pamphilis CW (2010). Identification of shared single copy nuclear genes in *Arabidopsis*, *Populus, Vitis* and *Oryza* and their phylogenetic utility across various taxonomic levels. BMC Evol Biol.

[11] De Smet R, Adams KL, Vandepoele K, Van Montagu MC, Maere S, Van de Peer Y (2013). Convergent gene loss following gene and genome duplications creates single-copy families in flowering plants. Proc Natl Acad Sci U S A.

[12] Small RL, Cronn RC, Wendel JF (2004). Use of nuclear genes for phylogeny reconstruction in plants. Aust Syst Bot.

[13] Wu F, Mueller LA, Crouzillat D, Pétiard V, Tanksley SD (2006). Combining bioinformatics and phylogenetics to identify large sets of single-copy orthologous genes (COSII) for comparative, evolutionary and systematic studies: a test case in the euasterid plant clade. Genetics.

[14] Li M, Wunder J, Bissoli G, Scarponi E, Gazzani S, Barbaro E, Saedler H, Varotto C (2008). Development of COS genes as universally amplifiable markers for phylogenetic reconstructions of closely related plant species. Cladistics.

[15] Emshwiller E, Doyle JJ (1999). Chloroplast-expressed glutamine synthetase (ncpGS): potential utility for phylogenetic studies with an example from *Oxalis* (Oxalidaceae). Mol Phylogenet Evol.

[16] Bulmer M (1991). The selection-mutation-drift theory of synonymous codon usage. Genetics.

[17] Akashi H (1994). Synonymous codon usage in Drosophila melanogaster: natural selection and translational accuracy. Genetics.

[18] Parmley JL, Hurst LD (2007). Exonic splicing regulatory elements skew synonymous codon usage near intron-exon boundaries in mammals. Mol Biol Evol.

[19] Zhou T, Weems M, Wilke CO (2009). Translationally optimal codons associate with structurally sensitive sites in proteins. Mol Biol Evol.

[20] Wright F (1990). The ‘effective number of codons’ used in a gene. Gene.

[21] Sharp PM, Li WH (1987). The codon Adaptation Index-a measure of directional synonymous codon usage bias, and its potential applications. Nucleic Acids Res.

[22] Xia X (2007). An improved implementation of codon adaptation index. Evol Bioinform Online.

[23] Gonçalves I, Duret L, Mouchiroud D (2000). Nature and structure of human genes that generate retropseudogenes. Genome Res.

[24] Ponger L, Duret L, Mouchiroud D (2001). Determinants of CpG islands: expression in early embryo and isochore structure. Genome Res.

[25] Duret L (2002). Evolution of synonymous codon usage in metazoans. Curr Opin Genet Dev.

[26] Tatarinova TV, Alexandrov NN, Bouck JB, Feldmann KA (2010). GC3 biology in corn, rice, sorghum and other grasses. BMC Genomics.

[27] Rao YS, Chai XW, Wang ZF, Nie QH, Zhang XQ (2013). Impact of GC content on gene expression pattern in chicken. Genet Sel Evol.

[28] Blencowe BJ (2006). Alternative splicing: new insights from global analyses. Cell.

[29] Xing Y, Lee C (2006). Alternative splicing and RNA selection pressure–evolutionary consequences for eukaryotic genomes. Nat Rev Genet.

[30] Keren H, Lev-Maor G, Ast G (2010). Alternative splicing and evolution: diversification, exon definition and function. Nat Rev Genet.

[31] The Angiosperm Phylogeny G (2009). An update of the Angiosperm Phylogeny Group classification for the orders and families of flowering plants: APG III. Bot J Linn Soc.

[32] Flagel LE, Wendel JF (2009). Gene duplication and evolutionary novelty in plants. New Phytol.

[33] Innan H, Kondrashov F (2010). The evolution of gene duplications: classifying and distinguishing between models. Nat Rev Genet.

[34] Xu J-H, Bennetzen JL, Messing J (2012). Dynamic gene copy number variation in collinear regions of grass genomes. Mol Biol Evol.

[35] Dopman EB, Hartl DL (2007). A portrait of copy-number polymorphism in *Drosophila melanogaster*. Proc Natl Acad Sci U S A.

[36] Jaillon O, Aury JM, Noel B, Policriti A, Clepet C, Casagrande A, Choisne N, Aubourg S, Vitulo N, Jubin C, Vezzi A, Legeai F, Hugueney P, Dasilva C, Horner D, Mica E, Jublot D, Poulain J, Bruyère C, Billault A, Segurens B, Gouyvenoux M, Ugarte E, Cattonaro F, Anthouard V, Vico V, Del Fabbro C, Alaux M, Di Gaspero G, Dumas V (2007). The grapevine genome sequence suggests ancestral hexaploidization in major angiosperm phyla. Nature.

[37] Tang H, Bowers JE, Wang X, Ming R, Alam M, Paterson AH (2008). Synteny and collinearity in plant genomes. Science.

[38] Tang H, Wang X, Bowers JE, Ming R, Alam M, Paterson AH (2008). Unraveling ancient hexaploidy through multiply-aligned angiosperm gene maps. Genome Res.

[39] Campbell WH, Gowri G (1990). Codon usage in higher plants, green algae, and cyanobacteria. Plant Physiol.

[40] Fennoy SL, Bailey-Serres J (1993). Synonymous codon usage in *Zea mays* L. nuclear genes is varied by levels of C and G-ending codons. Nucleic Acids Res.

[41] Sharp PM, Matassi G (1994). Codon usage and genome evolution. Curr Opin Genet Dev.

[42] Xing Y, Lee C (2005). Evidence of functional selection pressure for alternative splicing events that accelerate evolution of protein subsequences. Proc Natl Acad Sci U S A.

[43] Su Z, Wang J, Yu J, Huang X, Gu X (2006). Evolution of alternative splicing after gene duplication. Genome Res.

[44] Chen TW, Wu TH, Ng WV, Lin WC (2011). Interrogation of alternative splicing events in duplicated genes during evolution. BMC Genomics.

[45] Ner-Gaon H, Leviatan N, Rubin E, Fluhr R (2007). Comparative cross-species alternative splicing in plants. Plant Physiol.

[46] Barkman TJ, Simpson BB (2002). Hybrid origin and parentage of *Dendrochilum acuiferum* (Orchidaceae) inferred in a phylogenetic context using nuclear and plastid DNA sequence data. Syst Bot.

[47] Albach DC, Chase MW (2004). Incongruence in *Veroniceae* (Plantaginaceae): evidence from two plastid and a nuclear ribosomal DNA region. Mol Phylogenet Evol.

[48] Fehrer J, Gemeinholzer B, Chrtek J, Bräutigam S (2007). Incongruent plastid and nuclear DNA phylogenies reveal ancient intergeneric hybridization in *Pilosella hawkweeds* (*Hieracium*, Cichorieae, Asteraceae). Mol Phylogenet Evol.

[49] Felsenstein J (1978). Cases in which parsimony or compatibility methods will be positively misleading. Syst Bot.

[50] Phillips MJ, Delsuc F, Penny D (2004). Genome-scale phylogeny and the detection of systematic biases. Mol Biol Evol.

[51] Jeffroy O, Brinkmann H, Delsuc F, Philippe H (2006). Phylogenomics: the beginning of incongruence?. Trends Genet.

[52] Aguileta G, Marthey S, Chiapello H, Lebrun MH, Rodolphe F, Fournier E, Gendrault-Jacquemard A, Giraud T (2008). Assessing the performance of single-copy genes for recovering robust phylogenies. Syst Biol.

[53] Enright AJ, Van Dongen S, Ouzounis CA (2002). An efficient algorithm for large-scale detection of protein families. Nucleic Acids Res.

[54] Wang Y, Tang H, DeBarry JD, Tan X, Li J, Wang X, Lee T-h, Jin H, Marler B, Guo H (2012). MCScanX: a toolkit for detection and evolutionary analysis of gene synteny and collinearity. Nucleic Acids Res.

[55] Conesa A, Götz S, García-Gómez JM, Terol J, Talón M, Robles M (2005). Blast2GO: a universal tool for annotation, visualization and analysis in functional genomics research. Bioinformatics.

[56] Götz S, García-Gómez JM, Terol J, Williams TD, Nagaraj SH, Nueda MJ, Robles M, Talón M, Dopazo J, Conesa A (2008). High-throughput functional annotation and data mining with the Blast2GO suite. Nucleic Acids Res.

[57] Benzécri J-P (1992). Correspondence Analysis Handbook, Vol. 125.

[58] Xia X, Xie Z (2001). DAMBE: software package for data analysis in molecular biology and evolution. J Hered.

[59] Katoh K, Misawa K, Kuma K, Miyata T (2002). MAFFT: a novel method for rapid multiple sequence alignment based on fast Fourier transform. Nucleic Acids Res.

[60] Zhang Z, Li J, Zhao X-Q, Wang J, Wong GK-S, Yu J (2006). KaKs_Calculator: calculating Ka and Ks through model selection and model averaging. Genomics Proteomics Bioinformatics.

[61] Marquez Y, Brown JW, Simpson C, Barta A, Kalyna M (2012). Transcriptome survey reveals increased complexity of the alternative splicing landscape in *Arabidopsis*. Genome Res.

[62] Zhang G, Guo G, Hu X, Zhang Y, Li Q, Li R, Zhuang R, Lu Z, He Z, Fang X (2010). Deep RNA sequencing at single base-pair resolution reveals high complexity of the rice transcriptome. Genome Res.

[63] Stamatakis A (2006). RAxML-VI-HPC: maximum likelihood-based phylogenetic analyses with thousands of taxa and mixed models. Bioinformatics.

[64] Stamatakis A, Hoover P, Rougemont J (2008). A rapid bootstrap algorithm for the RAxML Web servers. Syst Bot.

