# Supplementary material for: Identification, characterization, and utilization of single copy genes in 29 angiosperm genomes
Source: BMC Genomics. 2014 Jun 21;15(1):504. doi: 10.1186/1471-2164-15-504 (PMC4092219; doi:10.1186/1471-2164-15-504)
Supplement: Supplementary file 3 — Additional file 3: Mann–Whitney U test for effective number of codons (Nc) and GC3. (DOCX 15 KB) [file 12864_2013_6214_MOESM3_ESM.docx]

**Additional file 3 Mann-Whitney *U* test for effective number of codons (Nc) and GC3.**

| Species | Mann–Whitney *U* test | |
| --- | --- | --- |
| *Aquilegia_coerulea* | SC_Nc<NSC_Nc(9.736e-040) | SC_GC3>NSC_GC3(0.049) |
| *Arabidopsis_lyrata* | SC_Nc<NSC_Nc(0.687) | SC_GC3<NSC_GC3(4.065e-005) |
| *Arabidopsis_thaliana* | SC_Nc<NSC_Nc(2.882e-015) | SC_GC3<NSC_GC3(5.435e-011) |
| *Brachypodium_distachyon* | SC_Nc>NSC_Nc(3.828e-017) | SC_GC3<NSC_GC3(1.064e-016) |
| *Brassica_rapa* | SC_Nc<NSC_Nc(3.133e-009) | SC_GC3<NSC_GC3(3.827e-009) |
| *Citrus_clementina* | SC_Nc<NSC_Nc(2.107e-016) | SC_GC3<NSC_GC3(1.873e-007) |
| *Carica_papaya* | SC_Nc<NSC_Nc(5.783e-018) | SC_GC3>NSC_GC3(1.233e-019) |
| *Capsella_rubella* | SC_Nc<NSC_Nc(5.541e-007) | SC_GC3<NSC_GC3(3.025e-021) |
| *Cucumis_sativus* | SC_Nc<NSC_Nc(1.486e-016) | SC_GC3<NSC_GC3(9.963e-005) |
| *Citrus_sinensis* | SC_Nc<NSC_Nc(2.137e-028) | SC_GC3<NSC_GC3(1.452e-005) |
| *Eucalyptus_grandis* | SC_Nc<NSC_Nc(9.703e-057) | SC_GC3<NSC_GC3(1.560e-247) |
| *Fragaria_vesca* | SC_Nc<NSC_Nc(1.331e-007) | SC_GC3>NSC_GC3(2.458e-078) |
| *Glycine_max* | SC_Nc<NSC_Nc(1.949e-061) | SC_GC3<NSC_GC3(2.369e-018) |
| *Gossypium_raimondii* | SC_Nc<NSC_Nc(1.696e-026) | SC_GC3<NSC_GC3(4.512e-006) |
| *Manihot_esculent* | SC_Nc<NSC_Nc(3.207e-007) | SC_GC3<NSC_GC3(9.056e-010) |
| *Mimulus_guttatus* | SC_Nc<NSC_Nc(1.111e-010) | SC_GC3<NSC_GC3(4.339e-048) |
| *Oryza_sativa* | SC_Nc>NSC_Nc(4.101e-078) | SC_GC3<NSC_GC3(2.113e-103) |
| *Prunus_persica* | SC_Nc>NSC_Nc(0.001) | SC_GC3<NSC_GC3(0.332) |
| *Populus_trichocarpa* | SC_Nc<NSC_Nc(6.089e-009) | SC_GC3<NSC_GC3(0.738) |
| *Phaseolus_vulgaris* | SC_Nc<NSC_Nc(0.004) | SC_GC3<NSC_GC3(0.009) |
| *Ricinus_communis* | SC_Nc<NSC_Nc(2.762e-180) | SC_GC3>NSC_GC3(0.000) |
| *Sorghum_bicolor* | SC_Nc>NSC_Nc(3.099e-063) | SC_GC3<NSC_GC3(1.945e-113) |
| *Setaria_italica* | SC_Nc>NSC_Nc(7.431e-034) | SC_GC3<NSC_GC3(4.224e-257) |
| *Solanum_lycopersicum* | SC_Nc<NSC_Nc(0.002) | SC_GC3>NSC_GC3(4.770e-124) |
| *Solanum_tuberosum* | SC_Nc<NSC_Nc(1.556e-007) | SC_GC3>NSC_GC3(1.569e-018) |
| *Theobroma_cacao* | SC_Nc<NSC_Nc(6.116e-036) | SC_GC3>NSC_GC3(5.747e-007) |
| *Thellungiella_halophila* | SC_Nc<NSC_Nc(0.021) | SC_GC3<NSC_GC3(1.661e-010) |
| *Vitis_vinifera* | SC_Nc<NSC_Nc(9.578e-064) | SC_GC3<NSC_GC3(2.275e-014) |
| *Zea_mays* | SC_Nc>NSC_Nc(7.252e-041) | SC_GC3<NSC_GC3(1.195e-102) |

SC_Nc: the Nc values of single copy genes; NSC_Nc: the Nc values of non-single copy genes; SC_GC3: the GC3 values of single copy genes; NSC_GC3: the GC3 values of non-single copy genes. Numbers inside parentheses represent the *p* values of two-tailed Mann–Whitney *U* Test.
