# Supplementary figures and images for: Identification, characterization, and utilization of single copy genes in 29 angiosperm genomes
Source: BMC Genomics. 2014 Jun 21;15(1):504. doi: 10.1186/1471-2164-15-504 (PMC4092219; doi:10.1186/1471-2164-15-504)

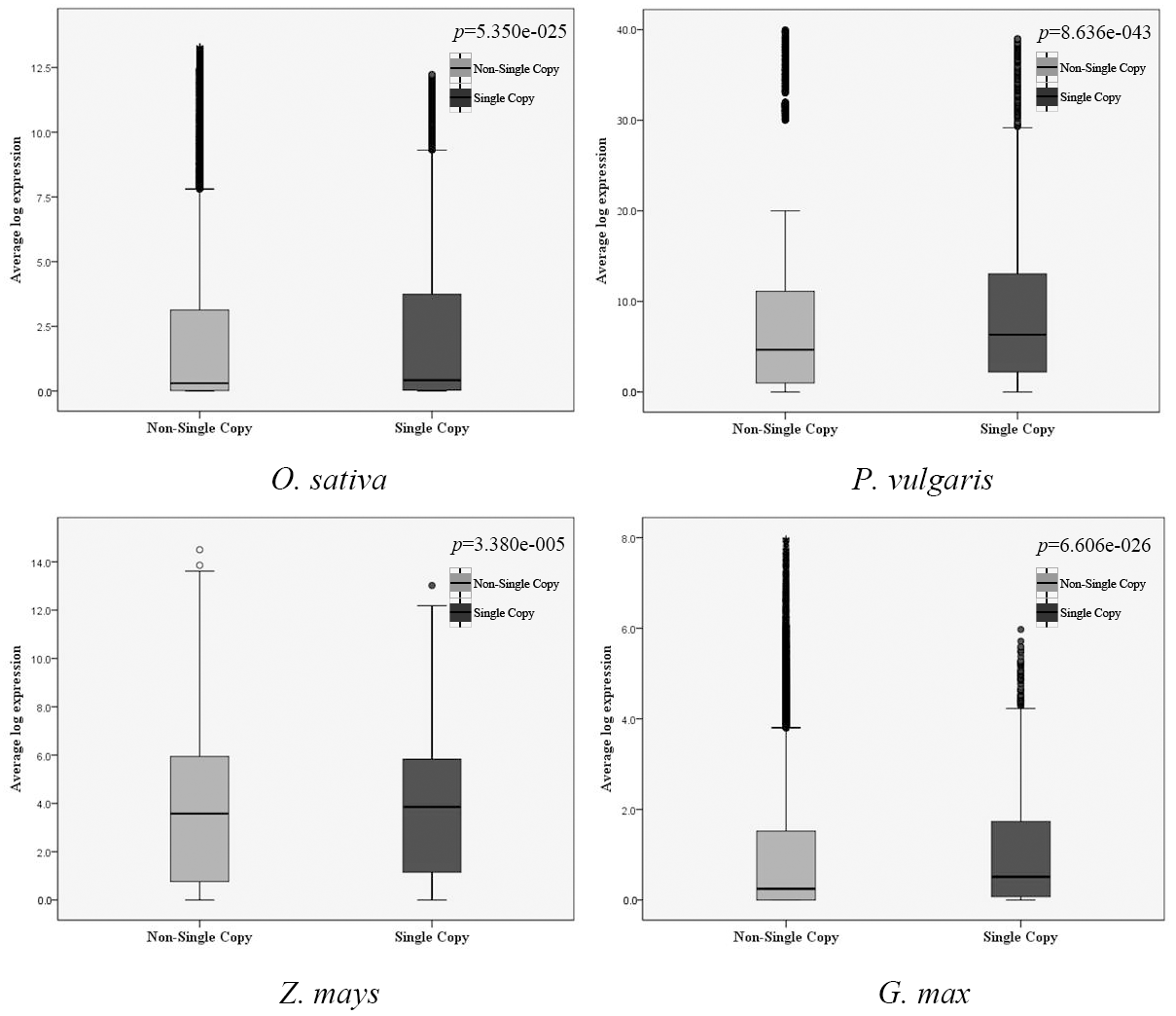

Supplement: Supplementary file 4 — Additional file 4: Average log expression levels for single copy genes in O. sativa , Z. mays , P. vulgaris and G. max. The significance between differences is calculated by the use of Mann–Whitney U test. (TIFF 4 MB) [file 12864_2013_6214_MOESM4_ESM.tiff]

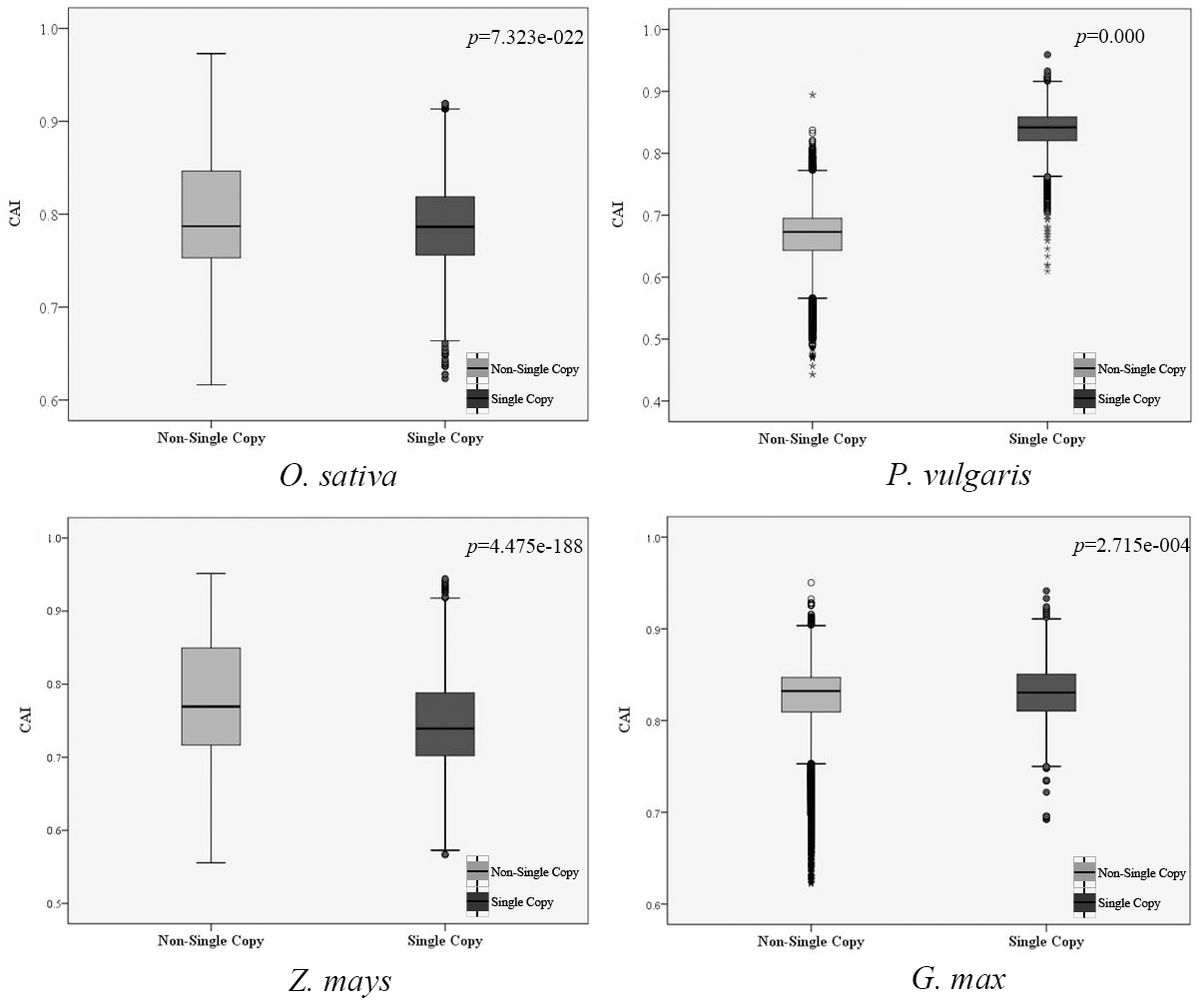

Supplement: Supplementary file 5 — Additional file 5: Codon Adaptation Index (CAI) for single-copy genes and no-single copy genes in O. sativa , Z. mays , P. vulgaris and G. max. The significance between differences is calculated by the use of Mann–Whitney U test. (TIFF 4 MB) [file 12864_2013_6214_MOESM5_ESM.tiff]

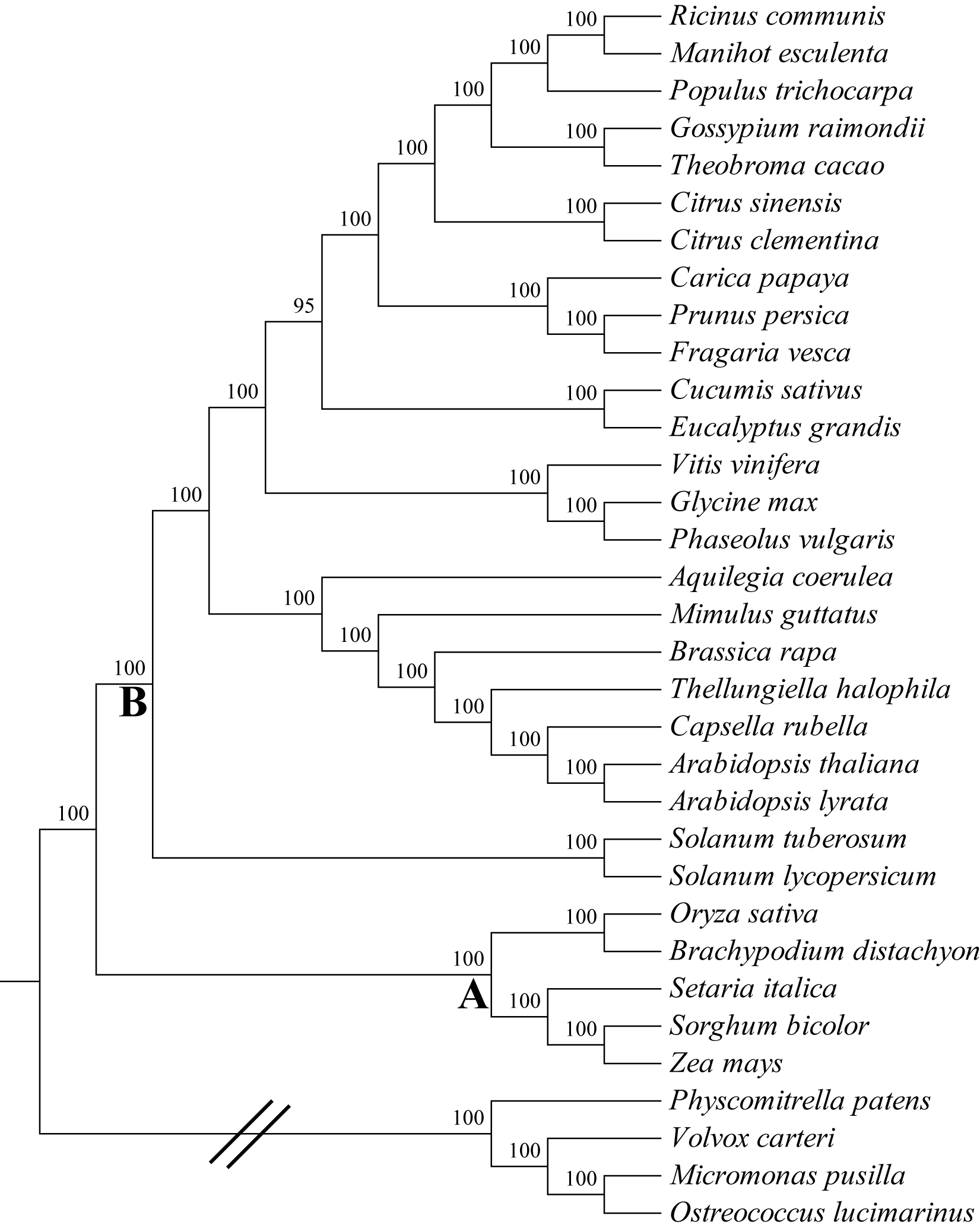

Supplement: Supplementary file 6 — Additional file 6: The ML phylogenetic tree bases on the 12 shared single copy genes including introns. Numbers above nodes are bootstrap proportions from 1000 pseudoreplicates. O. lucimarinus, M. pusilla, V. carteri and P. patens are used as outgroups. The letters embedded below nodes: A = Grass; B = Eudicots. The double slashes indicate outgroups. (ZIP 202 KB) [file 12864_2013_6214_MOESM6_ESM.zip › Additional_file_6.tif]

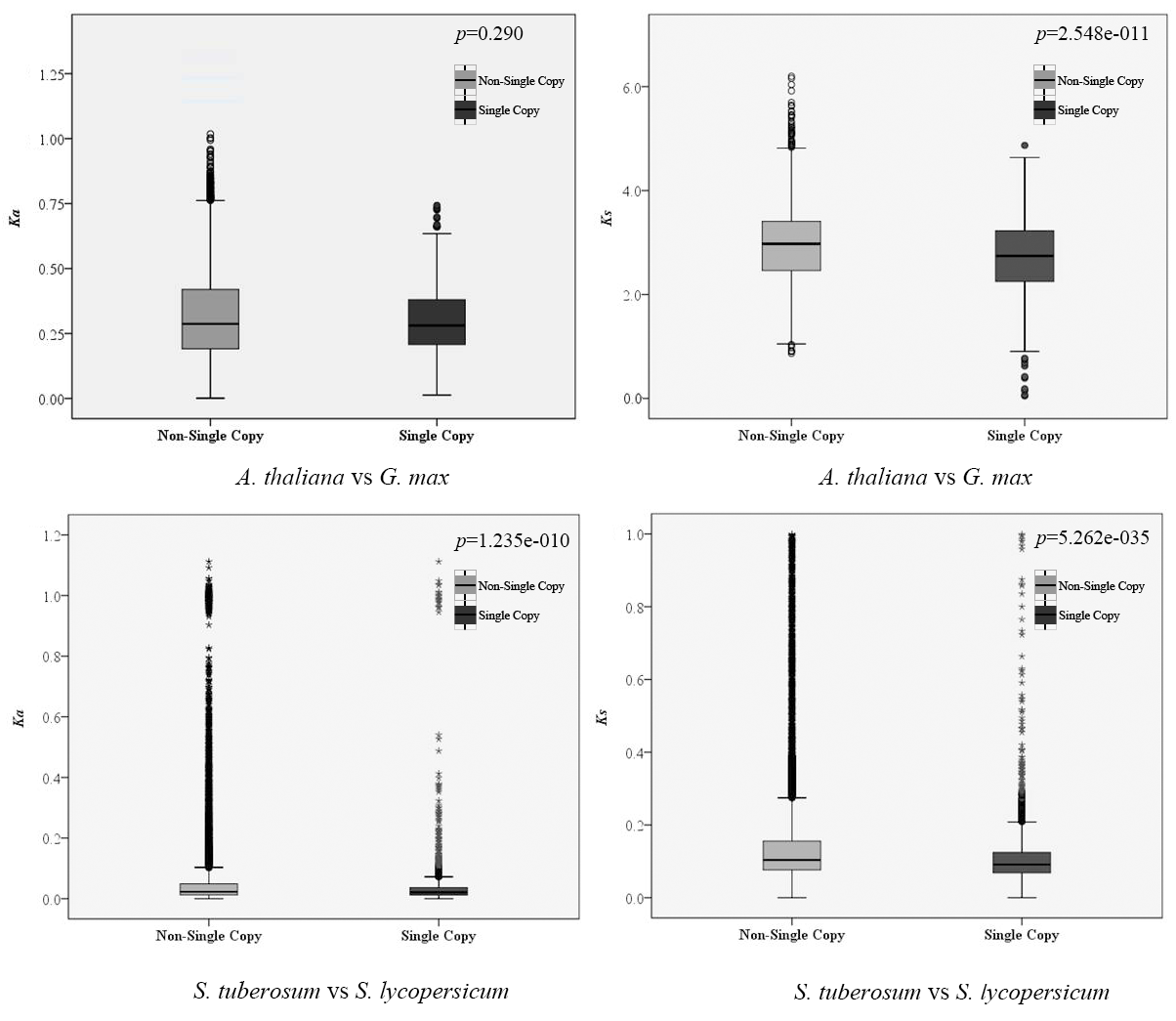

Supplement: Supplementary file 8 — Additional file 8: Average values of Ka and Ks for single copy genes in two species pairs. The significance between differences is calculated by the use of Mann–Whitney U test. (TIFF 4 MB) [file 12864_2013_6214_MOESM8_ESM.tiff]
