# Supplementary material for: Identification, characterization, and utilization of single copy genes in 29 angiosperm genomes
Source: BMC Genomics. 2014 Jun 21;15(1):504. doi: 10.1186/1471-2164-15-504 (PMC4092219; doi:10.1186/1471-2164-15-504)
Supplement: Supplementary file 7 — Additional file 7: Spearman correlation test comparing effective number of codons (Nc) versus GC3. (DOCX 15 KB) [file 12864_2013_6214_MOESM7_ESM.docx]

**Additional file 7 Spearman correlation comparing effective number of codons (Nc) versus GC3.**

| Species | Nc versus GC3 Spearman Test | |
| --- | --- | --- |
|  | Non-Single Copy | Single Copy |
| *Aquilegia coerulea* | (0.592,0.000) | (0.407,2.187e-196 ) |
| *Arabidopsis lyrata* | (0.298,0.000) | (0.288,9.454e-048 ) |
| *Arabidopsis thaliana* | (0.347,0.000) | (0.250,2.256e-061) |
| *Brachypodium distachyon* | (-0.792,0.000) | (-0.710,0.000) |
| *Brassica rapa* | (0.093,1.722e-073) | (0.101,2.203e-008) |
| *Capsella rubella* | (0.306,0.000) | (0.259,1.401e-056) |
| *Carica papaya* | (0.391,0.000) | (0.179,1.029e-060) |
| *Citrus clementina* | (0.429,0.000) | (0.382,0.3.382e-157) |
| *Citrus sinensis* | (0.415,0.000) | (0.345,2.034e-136) |
| *Cucumis sativus* | (0.468,0.000) | (0.279,2.168e-085) |
| *Eucalyptus grandis* | (-0.302,0.000) | (0.052,1.408e-005) |
| *Fragaria vesca* | (0.191,1.287e-216) | (0.026,0.043) |
| *Glycine max* | (0.298,0.000) | (0.250,3.036e-051) |
| *Gossypium raimondii* | (0.453,0.000) | (0.289,2.923e-074) |
| *Manihot esculent* | (0.481,0.000) | (0.395,7.899e-142) |
| *Mimulus guttatus* | (-0.063,1.373e-021) | (-0.017,0.286) |
| *Oryza sativa* | (-0.810,0.000) | (-0.600,0.000) |
| *Phaseolus vulgaris* | (0.308,0.000) | (0.244,8.372e-052) |
| *Populus trichocarpa* | (0.371,0.000) | (0.244,1.200e-041) |
| *Prunus persica* | (0.284,0.000) | (0.239,5.188e-052) |
| *Ricinus communis* | (0.254,6.046e-293) | (-0.266,8.354e-178) |
| *Setaria italica* | (-0.745,0.000) | (-0.322,4.584e-180) |
| *Solanum lycopersicum* | (0.534,0.000) | (0.281,2.793e-112) |
| *Solanum tuberosum* | (0.540,0.000) | (0.339,3.860e-151) |
| *Sorghum bicolor* | (-0.789,0.000) | (-0.527,0.000) |
| *Thellungiella halophila* | (0.203,2.609e-209) | (0.172,8.513e-026) |
| *Theobroma cacao* | (0.494,0.000) | (0.299,1.103e-139) |
| *Vitis vinifera* | (0.361,0.000) | (0.230,2.367e-068) |
| *Zea mays* | (-0.756,0.000) | (-0.484,0.000) |

Numbers inside parentheses represent the correlation coefficients and *p* values of two-tailed Spearman test, respectively.
